# Bax Inhibitor-1 preserves pancreatic β-cell proteostasis by limiting proinsulin misfolding and programmed cell death

**DOI:** 10.1038/s41419-024-06701-x

**Published:** 2024-05-14

**Authors:** Marina Blanc, Lama Habbouche, Peng Xiao, Cynthia Lebeaupin, Marion Janona, Nathalie Vaillant, Marie Irondelle, Jérôme Gilleron, Florent Murcy, Déborah Rousseau, Carmelo Luci, Thibault Barouillet, Sandrine Marchetti, Sandra Lacas-Gervais, Laurent Yvan-Charvet, Philippe Gual, Alessandra K. Cardozo, Béatrice Bailly-Maitre

**Affiliations:** 1 Institut National de la Santé et de la Recherche Médicale (Inserm) U1065, Université Côte d’Azur (UCA), Centre Méditerranéen de Médecine Moléculaire (C3M), Atip-Avenir, Fédération Hospitalo-Universitaire (FHU) Oncoage, Team “Hematometabolism and Metainflammation (HEMAMETABO), 06204 Nice, France; 2 Inflammation and Cell Death Signalling group, Signal Transduction and Metabolism Laboratory, Université libre de Bruxelles (ULB), Bruxelles, Belgique; 3 Degenerative Diseases Program, Sanford Burnham Prebys, La Jolla, CA 92037 USA; 4 Université Côte d’Azur, Institut National de la Santé et de la Recherche Médicale (Inserm) U1065, Adipo-Cible Research Study Group, Centre Méditerranéen de Médecine Moléculaire (C3M), Team «Insulin Resistance in Obesity and type 2 Diabetes», Nice, France; 5 Institut National de la Santé et de la Recherche Médicale (Inserm) U1065, Université Côte d’Azur, Centre Méditerranéen de Médecine Moléculaire (C3M), Team «Chronic Liver Diseases Associated with Obesity and Alcohol», Nice, France; 6 Institut National de la Santé et de la Recherche Médicale (Inserm) U1065, Université Côte d’Azur, Centre Méditerranéen de Médecine Moléculaire (C3M), Team «Metabolism, cancer and immune responses», Nice, France; 7 Université Côte d’Azur, Centre Commun de Microscopie Appliquée, CCMA, Nice, France

**Keywords:** Diabetes, Apoptosis

## Abstract

The prevalence of diabetes steadily increases worldwide mirroring the prevalence of obesity. Endoplasmic reticulum (ER) stress is activated in diabetes and contributes to β-cell dysfunction and apoptosis through the activation of a terminal unfolded protein response (UPR). Our results uncover a new role for Bax Inhibitor-One (BI-1), a negative regulator of inositol-requiring enzyme 1 (IRE1α) in preserving β-cell health against terminal UPR-induced apoptosis and pyroptosis in the context of supraphysiological loads of insulin production. *BI-1-*deficient mice experience a decline in endocrine pancreatic function in physiological and pathophysiological conditions, namely obesity induced by high-fat diet (HFD). We observed early-onset diabetes characterized by hyperglycemia, reduced serum insulin levels, β-cell loss, increased pancreatic lipases and pro-inflammatory cytokines, and the progression of metabolic dysfunction. Pancreatic section analysis revealed that *BI-1* deletion overburdens unfolded proinsulin in the ER of β-cells, confirmed by ultrastructural signs of ER stress with overwhelmed IRE1α endoribonuclease (RNase) activity in freshly isolated islets. ER stress led to β-cell dysfunction and islet loss, due to an increase in immature proinsulin granules and defects in insulin crystallization with the presence of Rod-like granules. These results correlated with the induction of autophagy, ER phagy, and crinophagy quality control mechanisms, likely to alleviate the atypical accumulation of misfolded proinsulin in the ER. In fine, BI-1 in β-cells limited IRE1α RNase activity from triggering programmed β-cell death through apoptosis and pyroptosis (caspase-1, IL-1β) via NLRP3 inflammasome activation and metabolic dysfunction. Pharmaceutical IRE1α inhibition with STF-083010 reversed β-cell failure and normalized the metabolic phenotype. These results uncover a new protective role for BI-1 in pancreatic β-cell physiology as a stress integrator to modulate the UPR triggered by accumulating unfolded proinsulin in the ER, as well as autophagy and programmed cell death, with consequences on β-cell function and insulin secretion.

**Figure:**
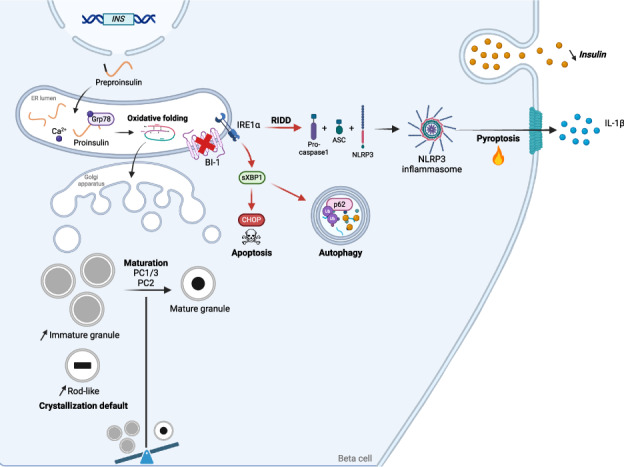
In pancreatic β-cells, *BI-1*^*–/–*^ deficiency perturbs proteostasis with proinsulin misfolding, ER stress, terminal UPR with overwhelmed IRE1α/XBP1s/CHOP activation, inflammation, β-cell programmed cell death, and diabetes.

In pancreatic β-cells, *BI-1*^*–/–*^ deficiency perturbs proteostasis with proinsulin misfolding, ER stress, terminal UPR with overwhelmed IRE1α/XBP1s/CHOP activation, inflammation, β-cell programmed cell death, and diabetes.

## Introduction

The International Diabetes Federation reports that diabetes affects 537 million adults worldwide and is predicted to rise to 783 million by 2045 [1]. Obesity leads to insulin resistance favoring metabolic diseases, including type 2 diabetes. Increasing our understanding of the molecular pathways behind the inhibition of insulin signal transduction and subsequent loss of functional β-cell mass remains a crucial challenge to define new therapeutic strategies. Growing experimental evidence suggests that endoplasmic reticulum (ER) stress may contribute to peripheral insulin resistance [2, 3] and play a role in premature pancreatic β-cell demise in diabetes in humans and preclinical animal models [4].

Pancreatic β-cells are responsible for insulin synthesis, storage, and secretion as the guardians of glucose homeostasis [5]. With insulin resistance, insulin production first increases, which overburdens the ER where proinsulin synthesis and folding occur. This overload causes ER stress and activates an evolutionarily conserved adaptative mechanism known as the unfolded protein response (UPR). The UPR is mediated by three transmembrane sensors: inositol-requiring enzyme 1 (IRE1α), PKR-like ER kinase (PERK), and activating transcription factor 6 (ATF6α) that bind intraluminally in unstressed conditions to the glucose-regulated protein and ER chaperone (BiP). Above a critical threshold of misfolded protein accumulation (e.g., proinsulin in β-cell ER), BiP dissociates from the sensors, thereby priming all branches for activation. With low levels of ER stress, a beneficial “physiological” induction of the UPR promotes β-cell homeostasis and adaptation to the demand for insulin production [3]. However, upon higher levels of ER stress as observed in obesity, a “pathophysiological” ER stress shifts the UPR to a terminal signaling cascade that induces apoptosis, notably via C/EBP homologous protein (CHOP), an apoptotic transcription factor whose expression is highly induced by ER stress in β-cells [6].

Genetic mutations in UPR components result in β-cell failure and diabetes in humans and mice [2, 3], implicating the UPR machinery in the proper functioning and survival of β-cells. Elevated ER stress, terminal UPR, apoptosis, and inflammation were reported in islets of diabetic patients and in animal models [3, 7]. Indeed, obese conditions perpetuated ER stress in β-cells, causing β-cell exhaustion and apoptosis that led to the progressive decline of insulin secretion [5]. Interestingly, misfolded proinsulin alone can cause diabetes in both humans in mice. In the *Akita* mouse model that recapitulates aspects of human monogenic diabetes, the Ins^2C96Y^ mutation resulted in misfolded proinsulin accumulation, ER stress, terminal UPR, and β-cell apoptosis, specifically by CHOP [6]. Since then, studies have strengthened the causal role of ER stress-mediated cell death and inflammation in diabetes [8, 9]. Discovering novel links in the switching process from adaptive to terminal UPR outputs in pancreatic β-cells undergoing ER stress is a potential strategy for diabetes treatment.

We postulated that Bax Inhibitor-1 (BI-1), an evolutionarily conserved ER transmembrane protein and apoptotic suppressor, could play a key role in the pathophysiology of diabetes [10]. BI-1 deletion may sensitize the pancreas to β-cell death and to diabetes, given that BI-1 is a negative regulator of IRE1α, the most conserved UPR sensor endowed with serine/threonine kinase and endoribonuclease (RNase) activities. IRE1α RNase activity leads to the unconventional splicing of XBP1 mRNA, translated into a transcription factor, sXBP1, that promotes ER protein folding and the adaptive UPR. Under chronic ER stress, sXBP1 activates target genes implicated in inflammation and apoptosis. IRE1α’s RNase activity also cleaves mRNAs and miRNAs through regulated IRE1α-dependent decay (RIDD) contributing to inflammation, metabolic perturbations, and programmed cell death [11]. IRE1α cleavage of miR17 leads to thioredoxin-interacting protein (TXNIP) accumulation, the activation of the NLRP3 inflammasome, and caspase-1-dependent interleukin (IL)-1β production, which can trigger pyroptosis that contributes to diabetes progression in humans and mice [12, 13].

We previously reported that the UPR induces NLRP3 inflammasome activation and hepatocyte death in steatohepatitis [14]. We next observed that BI-1 protects mice from an IRE1α-dependent metabolic derailment coupled with activation of the NLRP3 inflammasome, liver injury with hepatocyte loss attributed to programmed cell death in steatohepatitis [15]. Since the liver and pancreas are both major secretory organs, we hypothesized that BI-1 deficiency increases pancreatic β-cell sensitivity towards unrestricted IRE1α signaling-induced NLRP3 inflammasome activation, programmed cell death, and metabolic dysfunction, contributing to the onset of diabetes. Our results reveal an important role for BI-1 in secretory cell adaptation to ER stress in the context of supraphysiological loads of insulin production.

## Results

### BI-1 deficiency causes premature hyperglycemia through islet ER stress-dependent impaired insulin secretion

**Fig. 1:**
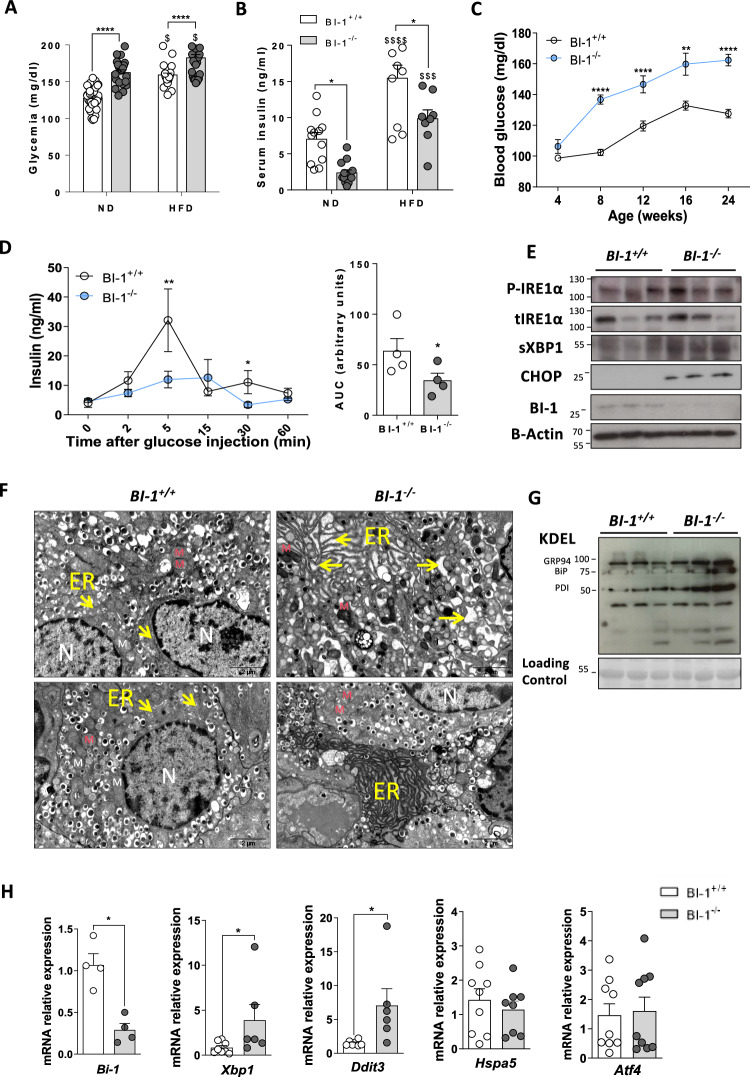
BI-1-deficient mice exhibit ER stress in pancreatic islets, resulting in hyperglycemia and reduced serum insulin levels in basal conditions. *BI-1*^*+/+*^ and *BI-1*^*–/–*^ mice were fed a normal diet (ND) for 6 months or a 3-month High-Fat Diet (HFD) starting at 3 months old. **A** Fed-state blood glucose levels at endpoint. *n* = 25–30 mice per group. **B** Serum insulin levels from respective genotype and diet. *n* = 8 mice per group. **C** Fed-state blood glucose levels above time. *n* = 15 mice per group. **D** Glucose-stimulated insulin secretion (GSIS) and Area Under the Curve (AUC) were performed. *n* = 3–4 mice per group. **E** Representative immunoblotting analysis of ER stress markers from total pancreatic protein lysates. *n* = 3 (out of 6) are shown per genotype. **F** Representative TEM images of pancreatic sections from respective genotype after ND. The nucleus is marked “N” and Mitochondria “M” in red. The yellow arrows point to the ER. Note the swelled and fragmented ER in *BI-1*^*–/–*^ compared to normal *BI-1*^*+/+*^ and the increased density of ER content in *BI-1*^*–/–*^ mice [Scale bar, 2 µm]. **G** Representative immunoblotting analysis of KDEL from total pancreatic protein lysates. *n* = 3 (out of 6) per genotype. **H** RT-qPCR analysis of pancreatic ER stress markers from *BI-1*^*+/+*^ and *BI-1*^*–/–*^ isolated islets. *n* = 8–9 mice per group. *P ≤ 0.05; **P ≤ 0.01; ***P ≤ 0.001. ****P ≤ 0.0001. $ represents differences in the same genotype with their own control.

Since we and others previously reported that BI-1, a master rheostat of ER stress, is invariably associated with the perturbation of glucose homeostasis [10, 15], we first confirmed our original observation [10] that *BI-1*^*−/−*^ mice exhibit perturbed glucose homeostasis after administration of an intraperitoneal bolus of glucose not only after high-fat diet (HFD) feeding but also on normal chow diet (ND; Fig. S1A and S1B). Unexpectedly, this occurred without significant changes in body weight or lipidaemia compared to control mice, even on HFD (Fig. S1C–F and S1G–J). Similar energy expenditure, respiratory quotient, food intake, and locomotor activity between genotypes measured by indirect calorimetry confirmed the absence of whole-body metabolic imbalance (data not shown). These findings suggested that impaired glucose homeostasis in *BI-1*^*−/−*^ mice could be due to defects in insulin secretion rather than peripheral insulin resistance. Consistent with this hypothesis, we observed that serum glucose levels were higher while insulin levels were lower in *BI-1*^*−/−*^ compared to *BI-1*^*+/+*^ mice in both ND and HFD conditions (Fig. 1A, B). Persistent hyperglycemia appeared at 8 weeks of age in ND-fed *BI-1*^*−/−*^ mice, resembling an early diabetes phenotype (Fig. 1C). This prompted us to directly evaluate glucose-stimulated insulin secretion in ND-fed *BI-1*^*−/−*^ mice. In contrast to *BI-1*^*+/+*^ mice, a glucose bolus did not increase plasma insulin levels in *BI-1*^*−/−*^ mice (Fig. 1D), suggesting that *BI-1*-deficient β-cells secrete insufficient amounts of insulin required to maintain normal glucose levels. PERK can regulate insulin secretion from the endocrine pancreas [16–19]. However, our immunoblotting analysis revealed similar levels of phosphorylated PERK in all mice (data not shown). In contrast, we observed significantly increased phosphorylated-IRE1α, sXBP1, CHOP protein expression, and absence of BI-1 in freshly isolated pancreas from *BI-1*^–/–^ compared to *BI-1*^*+/+*^ mice (Fig. 1E and Fig. S1K).

Transmission electron microscopy (TEM) analysis revealed that *BI-1*^–/–^ β-cells presented with swollen and fragmented ER, characteristic of ER stress (Fig. 1F). In line with sXBP1 induction [20], ER-resident proteins harboring the KDEL motif (e.g., BiP, GRP94 and PDI) displayed significantly higher expression in *BI-1*^*−/−*^ pancreases (Fig. 1G and Fig. S1L). Collectively, these data reveal that *BI-1* pancreatic deficiency promotes an ER stress response dependent on IRE1α signaling, which is consistent with our earlier observations in other tissues [15]. To delineate whether this effect was specific to β-cells, we freshly isolated them. *BI-1* deletion rendered β-cells more vulnerable to ER stress-dependent IRE1α RNase activity shown by mRNA induction of *Xbp1* and *Ddit3* (encoding CHOP), but not *Hspa5* (encoding BIP) or *Atf4* (Fig. 1H)*.* Hence, we speculate that hyperglycemia in *BI-1*^–/–^ mice may be primarily due to an ER stress-dependent dysfunction of the endocrine pancreas to secrete adequate amounts of insulin at an early age or under diet-induced diabetic conditions.

### BI-1 deletion leads to pancreatic β-cell loss

**Fig. 2:**
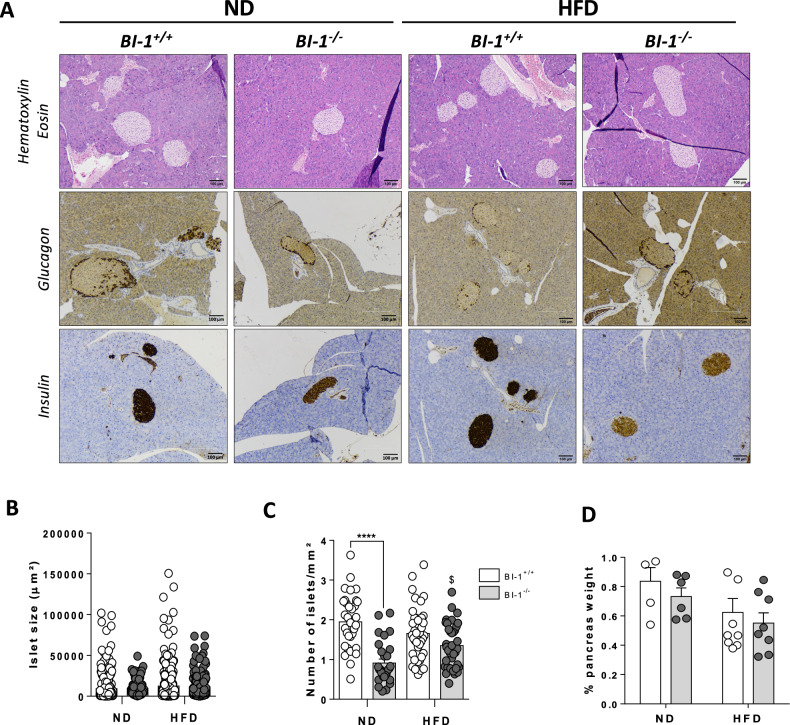
BI-1 deletion impairs pancreatic β-cell mass. *BI-1*^*+/+*^ and *BI-1*^–/–^ mice were treated as in Fig. 1. **A** Representative hematoxylin and eosin, insulin, and glucagon staining of pancreatic sections from *BI-1*^*+/+*^ and *BI-1*^*–/–*^ mice fed ND or HFD as in Fig. 1. [Scale bar, 100 µm]. *n* = 8–10 mice per group. **B** Quantification of pancreatic islet size was obtained from insulin staining of pancreatic sections from *BI-1*^*+/+*^ and *BI-1*^*–/–*^ mice fed ND or HFD. *n* = 8–10 mice per genotype. **C** Quantification of the number of islets per mm² were obtained from *BI-1*^*+/+*^ and *BI-1*^*–/–*^ mice fed ND or HFD. *n* = 8–10 mice per group. **D** Pancreas weight expressed as % body weight from *BI-1*^*+/+*^ and *BI-1*^*–/–*^ mice under ND and HFD conditions was measured. *n* = 6–8 mice per group. *P ≤ 0.05; **P ≤ 0.01; ***P ≤ 0.001. ****P ≤ 0.0001. $ represents differences in the same genotype with their own control.

Histological analysis of pancreatic sections revealed no major structural differences in hematoxylin and eosin (H&E)-stained exocrine pancreases between *BI-1*^+/+^ and *BI-1*^–/–^ mice fed either ND or HFD. To evaluate the balance between α- and β-cell adaptation in pancreatic islets, we performed immunohistochemistry for glucagon and insulin, respectively (Fig. 2A). Glucagon immunostaining revealed no differences in pancreatic α-cell mass, location, or repartition between *BI-1*^+/+^ and *BI-1*^–/–^ mice on either ND or HFD (Fig. 2A), suggesting no glucagon insufficiency in the endocrine pancreas of *BI-1*^–/–^ mice. However, anapathological examination of H&E and insulin-positive staining revealed reduced pancreatic islet mass in these animals in both feeding conditions (Fig. 2A) Quantification of pancreatic islet size, and number confirmed that *BI-1* deficiency reduced functional β-cell mass by fewer islets per area, independently of islet size differences (Fig. 2B, C) or percentage of pancreatic weight (Fig. 2D). Hence, a deficiency in *BI-1* resulting in unrestrained IRE1α/XBP1 signaling may predispose to pancreatic islet loss, a hallmark of early-onset diabetes that can be exacerbated upon diet-induced diabetes.

### Lack of BI-1 and induction of UPR signalling promotes autophagy, inflammatory response, and ultimately pancreatic-β-cell death resembling monogenic-like diabetes

While WT (*BI-1*^+/+^) mice presented mature and immature insulin granules typical of healthy pancreatic β-cells analyzed by TEM (Figure S2Aa), *BI-1*^–/–^ β-cells revealed an atypical accumulation of autophagosomes (Fig. S2Ab), and lysosomes (Fig. S2Afg). We also observed ultrastructural features of ER-phagy (Fig. S2Acdj), crinophagy (Fig. S2Ae), and mitophagy (Fig. S2Ai) in β-cells of *BI-1*^–/–^ but not WT mice, highlighting a mobilization of quality control mechanisms to cope with ER stress.

We then analyzed the levels of autophagy markers in pancreatic lysates from *BI-1*^–/–^ mice. A higher amplitude of LC3-II conversion [21, 22] and a slight increase in basal p62/SQSTM1 expression was observed in *BI-1*^*−/−*^ mice by immunoblotting analysis (Figs. S2B and S3A). Accordingly, a significantly higher expression of the Atg5–Atg12 complex and a stronger accumulation of total ubiquitinylated proteins on Lysin 63 (K63) were observed in *BI-1*^–/–^ compared to WT pancreas (Figs. S2C and S3B). *BI-1*^*–/–*^ mice exhibited reduced expression of the mitophagy markers Parkin and COX4 (Figs. S2D and S3C), indicating increased mitochondrial degradation through mitophagy.

**Fig. 3:**
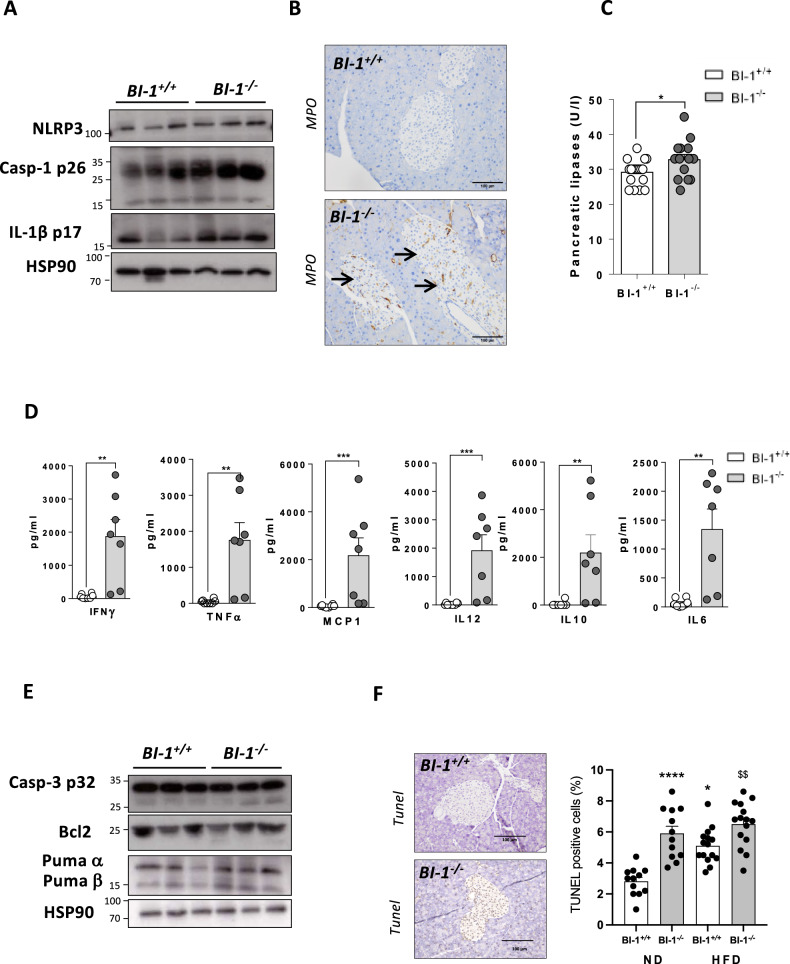
BI-1 loss increases pancreatic β-cell death through apoptosis and NLRP3 inflammasome activation. *BI-1*^*+/+*^ and *BI-1*^*–/–*^ mice were fed a 6-month ND. **A** Representative immunoblotting analysis of NLRP3 inflammasome markers and substrate namely NLRP3, active-caspase-1 and IL-1β from total pancreatic protein lysates. *n* = 3 (from 6 mice per genotype) are presented. **B** Representative myeloperoxidase (MPO) staining for neutrophil infiltration (arrows) of pancreatic sections are shown [Scale bar, 100 µm]. *n* = 3–5 mice per group. **C** Serum pancreatic lipase levels are presented. *n* = 10–12 mice per group. **D** Relative serum cytokine levels evaluated by flow cytometry analysis. *n* = 7–8 mice per group. **E** Immunoblotting analysis of apoptotic markers namely Caspase-3, Bcl2, and Puma (α and β), in total pancreatic protein lysates. Representative *n* = 3 out of 6 mice per genotype are shown. **F** Representative pictures of TUNEL staining from *BI-1*^*+/+*^ and *BI-1*^*–/–*^ pancreatic sections under steady state and HFD. Number of apoptotic TUNEL-positive β-cells. *n* = 3 mice. *P ≤ 0.05; **P ≤ 0.01; ***P ≤ 0.001, ****P ≤ 0.0001. $ represents differences in the same genotype with their own control.

Because autophagy, inflammation, and programmed β-cell death are interconnected to preserve cell homeostasis, we next evaluated the inflammatory response in *BI-1*^–/–^ mice, specifically NLRP3 inflammasome activation. *BI-1*^–/–^ pancreases presented significantly increased expression of NLRP3 protein, correlating with higher levels of active-caspase-1 and IL-1β than WT pancreases (Fig. 3A and Fig. S3D). Furthermore, pancreatic sections from *BI-1*^–/–^ mice had more myeloperoxidase (MPO)-positive cells compared to WT mice, reflecting abnormal inflammatory myeloid cell infiltration (Fig. 3B), as reported in diabetic patients [7]. This was associated with elevated serum levels of pancreatic lipases (Fig. 3C), and inflammatory markers (i.e, IFNγ, TNFα, MCP-1, IL-12, IL-10, and IL-6) (Fig. 3D), reflecting pancreatic injury in *BI-1*^–/–^ mice. This prompted us to evaluate the impact of *BI-1* deficiency on programmed cell death in pancreatic islets. Immunoblotting analysis revealed significantly increased cleaved caspase-3 and pro-apoptotic Puma, a BH3-only protein that inhibits the anti-apoptotic BCL2, in *BI-1*^–/–^ mice (Fig. 3E and Fig. S3E). Cells undergoing pyroptosis, like apoptosis, incur DNA damage and become positive by terminal deoxynucleotidyl transferase dUTP nick-end labeling (TUNEL) assay. Pancreatic sections from *BI-1*-deficient mice had a greater number of TUNEL+ β-cells compared to WT mice (Fig. 3F), which persisted after HFD, with no difference in β-cell proliferation (Ki-67+; not shown).

**Fig. 4:**
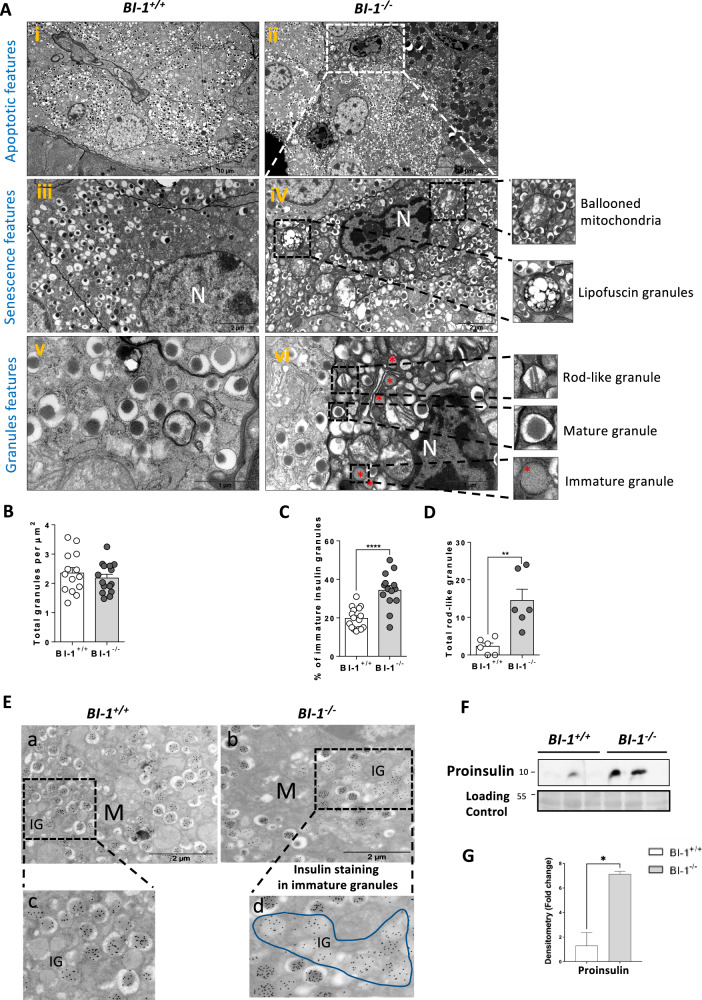
BI-1 deletion causes defects in insulin maturation. *BI-1*^*+/+*^ and *BI-1*^–/–^ mice were fed a 6-month ND. **A** Representative TEM images of pancreatic sections with β-cells and insulin granule morphology are shown [Scale bar, 10 µm, 2 µm, 1 µm]. Suffering pancreatic β-cells are represented in a white rectangle. N: nucleus. Lipofuscin granules and altered mitochondria are in black rectangle. Increased magnification on different insulin granules state shows immature (red asterisks), mature and rod-like granules. *n* = 4 mice. Respective quantification of **B** total mature insulin granules, **C** % of immature insulin granules, **D** total rod-like granules. Quantification was performed from 10 to 15 images per *n*; *n* = 4 mice. **E** The immunogold staining looks stronger in *BI-1* deficient mice insulin granules (b, d) than in WT (a, c), particularly in immature granules (IG) which clearly appear more numerous (surrounded by a line in *BI-1* deficient mice). **F** Representative immunoblotting analysis of proinsulin pancreatic protein lysates from WT and *BI-1*^*−/−*^*. n* = 3 (out of 6) mice are shown per genotype. **G** Immunoblotting quantification of proinsulin. *P ≤ 0.05; **P ≤ 0.01; ***P ≤ 0.001. ****P ≤ 0.0001. $ represents differences in the same genotype with their own control.

Further investigation of the ultrastructural pancreatic β-cell phenotype revealed well-preserved α-cells in pancreatic sections from *BI-1*-deficient mice (Fig. 4Ai)*.* However, pancreatic sections from *BI-1*-deficient animals presented apoptotic features specifically in β-cells, with chromatin and cytosol condensation, altered mitochondria (Fig. 4Aii, iv), and the presence of lipofuscin granules, a marker of senescence (Fig. 4Aiv). Overall, *BI-1*-deficient pancreases exhibited higher levels of ER stress-induced adaptive autophagy and inflammasome activation that are most likely the culprit of apoptotic and pyroptotic cell death and subsequent loss of β-cells, as observed in monogenic diabetes.

### Increased immature insulin granules with higher relative content of proinsulin in pancreatic islets is associated with β-cell dysfunction in BI-1-deficient mice

Impaired insulin secretion was previously linked to excessive β-cell death [23] in diabetes and an imbalance between efficient mature insulin granule formation with a compensatory accumulation of non-functional immature insulin granules [24–26]. Typical mature insulin granules exhibit a dense homogenous core with a well-defined halo, whereas immature granules exhibit an empty or lighter core without a defined halo (Fig. 4Av, vi). No differences in the number of total insulin granules per area were observed between *BI-1*^–/–^ and WT β-cells (Fig. 4B), while the percentage of immature insulin granules revealed a nearly two-fold significant increase in *BI-1*^–/–^ β-cells (Fig. 4C). In addition, we detected the presence of rod-like granules (Fig. 4D), ultrastructural characteristics resulting from insulin crystallization and packaging defects [27, 28], in significantly greater abundance in *BI-1*^–/–^ versus WT β-cells. These observations were corroborated by insulin immunogold staining, which was stronger in insulin granules of *BI-1*^–/–^ mice showing more numerous immature granules (Fig. 4Eb, d; outlined) than *BI-1*^+/+^ mice (Fig. 4Ea, c). Insulin immunogold staining further confirmed the insulin granules were located in autolysosomes (Fig. S2E). Finally, we reported a significantly stronger expression of proinsulin protein in *BI-1*^*−/−*^ compared to WT pancreases (Fig. 4F, G). Pancreatic β-cell loss in *BI-1*-deficient mice is most likely driven by misfolded insulin accumulation, responsible for stressed β-cells and progressive pancreatic β-cell identity loss, as shown by ultrastructural signs of senescence and apoptosis.

### BI-1 deletion increases ER stress-induced inflammasome activation and cell death in human and mouse β-cells

**Fig. 5:**
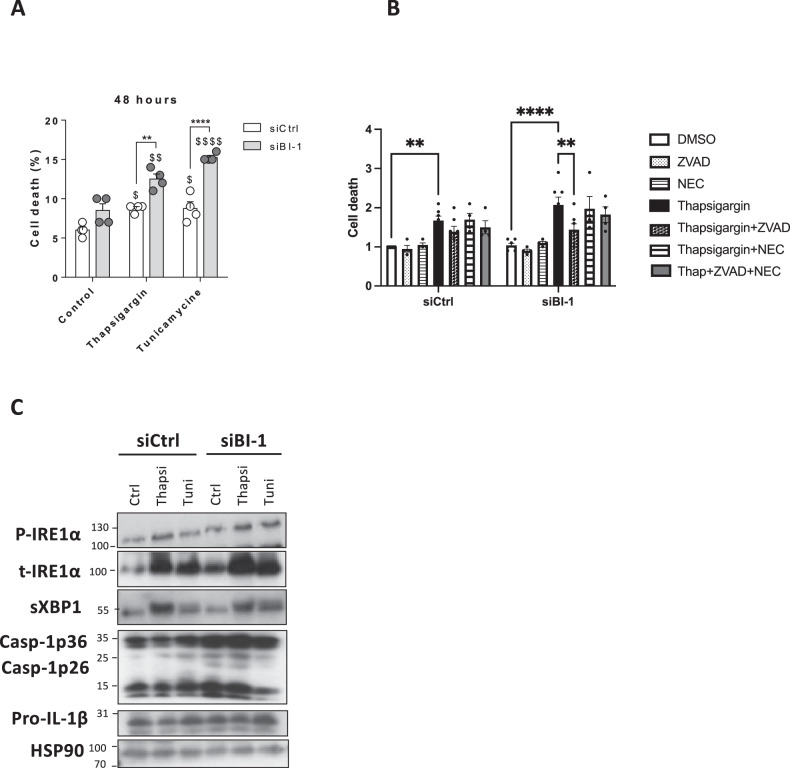
BI-1 deletion increases cell death and leads to ER stress and activated inflammasome markers in human β-cells. **A** Cell death was quantified in the human β−cell line EndoC-βH1 cells transfected with control (siCrtl) or *BI-1* siRNA (siBI-1) in response to chemical ER stress (Thapsigargin 1 μM; Tunicamycin 5 μg/ml) compared to normal media. *n* = 4. $*P* ≤ 0.05; $$*P* ≤ 0.01; $$$*P* ≤ 0.001. $$$$*P* ≤ 0.0001. $ represents differences with control. *Represents differences with indicated treated conditions, **P ≤ 0.01; ****P ≤ 0.0001. **B** Cell death was quantified in EndoC-βH1. The concentrations of chemicals were used as following: z-VAD-FMK (50 μmol/L, 30 min pre-incubation), Necrostatin-1 (Nec-1) (10 μmol/L, thapsigargin (1 μmol/L), or an equal volume of DMSO (Sigma-Aldrich). $ represents differences with control. *Represents differences with indicated treated conditions, **P ≤ 0.01; ****P ≤ 0.0001. *n* = 4–7. **C** Western blotting analysis of phospho-IRE1α, sXBP1, active-caspase-1, and pro-IL-1β protein levels assessed from EndoC-βH1 cells transfected with control or *BI-1* siRNA prior to treatment (*n* = 4–7).

The relevance of *BI-1* loss on IRE1α-dependent inflammasome activation and cell death was investigated in the human β-cell line EndoCβH1 in response to chemical ER stress agents, i.e., tunicamycin and thapsigargin. Like our observations in the *BI-1*^–/–^ pancreases*, BI-1*-silenced human β-cells exhibited increased ER stress-induced cell death compared to control β-cells (Fig. 5A). Because effector caspases are required for the execution phases of apoptosis, we used the pan caspase inhibitor N-Benzyloxycarbonyl-Val-Ala-Asp(O-Me) fluoromethyl ketone (Z-VAD-fmk). ER stress-induced cell death was suppressed with Z-VAD-fmk, suggesting apoptosis (Fig. 5B). *BI-1*-silenced human β-cells also displayed activated IRE1α signaling with phospho-IRE1α and sXBP1 protein accumulation (Fig. 5C and Fig. S4A, B) and inflammasome activation, shown by higher active-caspase-1(p36) protein levels and a tendency to increase the pro-IL-1β protein levels, when treated with ER stressors (Fig. 5C and Fig. S4C–E). Mimicking inflammation with various cytokine cocktails in the media was insufficient to promote cell death in *BI-1*-silenced β-cells (data not shown), indicative of inflammation as a consequence rather than cause of *BI-1*-dependent ER stress. To complete these data, and in particular, to explore the role of necroptosis and its potential regulation by BI-1, we conducted experiments using Necrostatin-1 (NEC), a specific inhibitor of necroptosis (Fig. 5B). The use of Nec-1 failed to rescue cell death in our experimental conditions, suggesting that necroptosis is not involved.

These data were confirmed in the murine β-cell line MIN6. Compared to control β-cells, *BI-1*-silenced murine β-cells displayed increased ER stress-induced cell death. Thapsigargin-induced cell death was significantly suppressed with the broad effector caspase inhibitor Q-Val-Asp fluoromethyl ketone (QVD-fmk), supporting the concept that BI-1 protects against ER stress-induced apoptosis in these cells (Fig. S5A).

We next tested the relevance of using Ac-YVAD-fmk (Ac-Tyr-Val-Ala-Asp- fluoromethyl ketone), a caspase-1 inhibitor in our experimental conditions. Thapsigargin-induced cell death was significantly reduced with YVAD-fmk, suggesting that BI-1 seems cytoprotective against ER stress-induced-caspase-1- and-inflammasome activation in these cells. Finally, combining QVD-fmk and Ac-YVAD-fmk completely rescued the thapsigargin-induced cell death in MIN6 (Fig. S5B). In all experiments, after 48 h of silencing, we observed markedly reduced BI-1 protein levels (Fig. S5C) with mirroring increases in XBP1 mRNA levels (Fig. S5D), supporting the notion that a reduction in BI-1 might favor enhanced ER stress, through IRE1α, in MIN6 cells.

Overall, these results strengthened the model that *BI-1*-deletion makes β-cells more vulnerable to ER stress-induced-programmed cell death through apoptosis and pyroptosis.

Together, these findings strongly suggest a conserved function of BI-1 in mammalian β-cells with human pathological relevance.

### Pharmaceutical IRE1α inhibition reverses β-cell failure and associated metabolic disorders

**Fig. 6:**
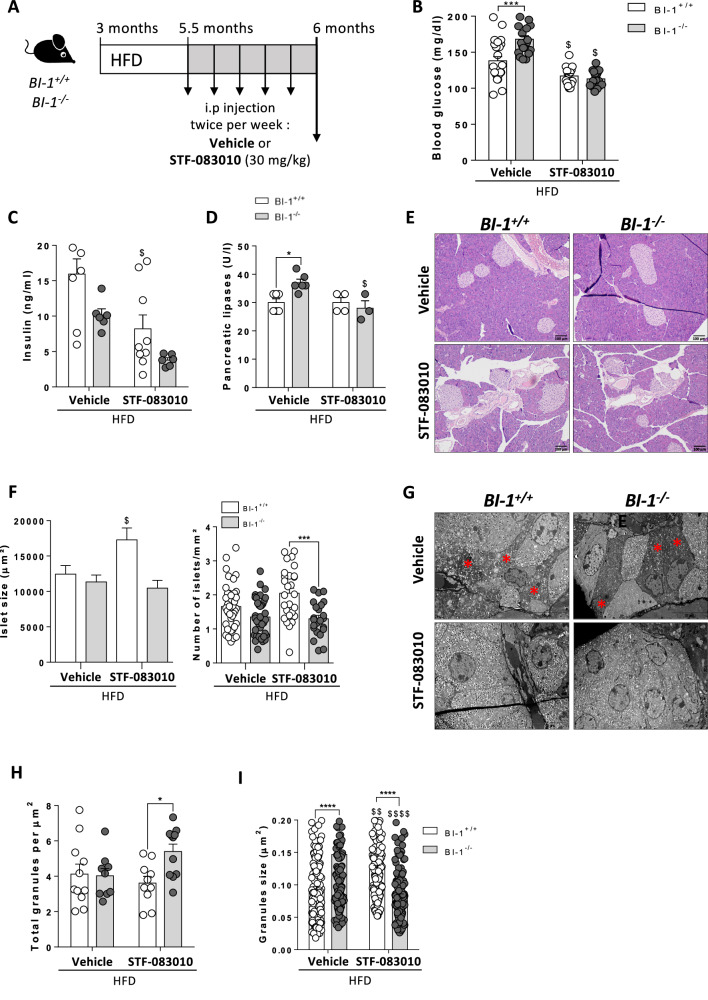
IRE1α inhibition corrects pancreatic injury and associated metabolic disorders in BI-1 WT and KO mice. **A** Protocol timeline for vehicle (kolliphor 16%) or STF-083010 injection (30 mg/kg) in *BI-1*^*+/+*^ and *BI-1*^*–/–*^ mice twice a week for 2 weeks before sacrifice during a 3-month HFD starting at 3 months old. **B** Blood glucose levels in HFD-fed-*BI-1*^*+/+*^ and *BI-1*^*–/–*^ mice injected with STF-083010 or vehicle. *n* = 15–25 mice per group. **C** Relative insulin levels in the sera. *n* = 8 mice. **D** Serum pancreatic lipases levels from HFD-*BI-1*^*+/+*^ and *BI-1*^*–/–*^ mice injected with STF-083010 or not (vehicle) at the end of the HFD-diet. *n* = 3–6 per group. **E** Representative images of hematoxylin and eosin staining from HFD-*BI-1*^*+/+*^ and *BI-1*^*–/–*^ pancreatic sections [Scale bar, 100 µm]. *n* = 6–8 mice per group. **F** Islet size was quantified. *n* = 6–8 mice per group. Quantification of number of islets per mm². *n* = 6–8 mice. **G** Representative TEM images of pancreatic sections from HFD-fed *BI-1*^*+/+*^ and *BI-1*^*–/–*^ injected with STF-083010 or vehicle. *n* = 3–4 mice per group. [Scale bar, 10 µm]. **H** Quantification of the total granules per µm² is measured from TEM images from *BI-1*^*+/+*^ and *BI-1*^*–/–*^ mice pancreatic sections treated with STF-083010 or vehicle. *n* = 3–4 mice per group. **I** Insulin granule size was measured from representative TEM images in HFD-fed mice treated with STF-083010 or vehicle (10-15 pictures per n, *n* = 3–4 mice per group). *P ≤ 005; **P ≤ 0.01; ***P ≤ 0.001. ****P ≤ 0.0001. $ represents differences in the same genotype with their own control.

Given the regulatory function of BI-1, we hypothesized that inhibiting IRE1α activity may protect against β-cell failure and diabetes. Thus, we evaluated the potential of treating mice in a regression model: *BI-1*^*–/–*^ mice on a 3-month HFD to induce IRE1α RNase activity-dependent ER stress, β-cell dysfunction, and diabetes were treated with an inhibitor of IRE1α RNase activity, STF-083010, twice a week during the last two weeks of diet (Fig. 6A). Body weight gain and pancreas weight were similar in both *BI-1*^*–/–*^ and WT mice, irrespective of STF-083010 or vehicle treatment (data not shown). Nevertheless, targeting IRE1α RNase activity normalized blood glucose concentrations in both *BI-1*^*–/–*^ and WT mice fed HFD (Fig. 6B), reduced hyperinsulinemia (Fig. 6C) and pancreatic lipase levels (Fig. 6D), reflecting an improvement in glucose homeostasis and β-cell function. Consistently, STF-083010 significantly decreased myeloid cell infiltration (Fig. S6A) and sera pro-inflammatory cytokine levels (Fig. S6B) in *BI-1*^*–/–*^ mice. STF-083010 limited pancreatic injury and associated inflammation caused by *BI-1* deficiency. Intriguingly, STF-083010 did not rescue the reduction in islet number observed in HFD-fed *BI-1*^–/–^ mice, although this treatment slightly increased islet size in diabetic HFD-fed control animals (Fig. 6E, F). We next explored whether inhibition of IRE1α RNase activity could improve β-cell function in *BI-1*^–/–^pancreas. Analysis of pancreatic sections by TEM revealed that STF-083010 decreased the ultrastructural evidence of β-cell apoptosis (chromatin condensation and cell shrinkage) and reduced the presence of altered mitochondria and autophagolysosomes (Fig. 6G). STF-083010 normalized the ultrastructural signs of ER stress, with restored regularly spaced stacks of ER sheets (Fig. 6G) β-cell function improvement after STF-083010 treatment in the pancreases of HFD-fed *BI-1*^*–/–*^ mice was associated with increased total insulin granules (Fig. 5H), likely due to improved granule size and functionality since STF-083010 normalized granule size originally hypertrophied in HFD-*BI-1* KO mice (Fig. 5I). Thus, we uncovered a potential novel role for BI-1 in regulating IRE1α’s RNase activity and ER stress in the balance between pancreatic inflammation and apoptosis with consequences on β-cell function and insulin secretion.

## Discussion

In the natural history of diabetes, the progressive decline in β-cell function precedes the loss of β-cell functional mass; however, the underlying mechanisms are unclear but may involve the ER stress response. Even if the upstream stresses differ between types 1 and 2 diabetes, with an autoimmune attack against the β-cells or the development of peripheral insulin resistance, respectively, the downstream outcome would be similar. The remaining β-cells would be overworked and experience critical ER stress levels resulting in β-cell dysfunction and death through terminal UPR activation. Our results bring new insights into the protective and adaptative role of BI-1 against the detrimental outputs of the UPR during ER stress in β-cells.

We revealed that *BI-1* deficiency causes a progressive decline in endocrine pancreatic function in physiological and pathophysiological conditions. A close correlation was observed between hyperglycemia, reduced serum insulin levels, loss of β-cell mass, and metabolic disorders in *BI-1-*deficient mice, resembling early-onset diabetes. Both the endocrine and exocrine pancreas developed normally, indicating that endocrine dysfunction is acquired postnatally. Hence, we found that *BI-1* deletion leads to ER stress in β-cells, coupled with β-cell dysfunction due to increased immature proinsulin granules and defective insulin crystallization with Rod-like granules. These observations correlated with autophagy induction, likely to alleviate the accumulation of misfolded proinsulin in the ER and cope with ER stress. *In fine*, in *BI-1-*deficient pancreatic islets, sustained IRE1α signaling triggers programmed β-cell death with the activation of apoptosis and pyroptosis through NLRP3 activation.

In *BI-1-*deficient β-cells, the inability to properly fold large, secretory loads may cause accumulation of unfolded proinsulin within the ER, explaining the vulnerability to ER stress-induced-programmed cell death. Ultrastructural analysis of *BI-1*^–/–^ pancreas confirmed striking abnormal changes in ER morphology, a hallmark of ER stress and UPR activation. While α-cells looked morphologically normal, β-cells from *BI-1*^–/–^ mice presented massive ER lumen dilation with accumulation of large quantities of electron-dense material, suggesting ER stress with deposition of unfolded proteins in the ER. Similar observations were reported in both *Akita* and *Munich* mouse models, in which the insulin mutations cause proinsulin misfolding-induced terminal UPR [6], β-cell failure, and neonatal diabetes [29–31]. *Akita*-like insulin mutations cause rare infantile diabetes described in humans [29]. *Eif2ak3*^*–/–*^ mice developed similar phenotypes with hyperglycemia, hypoinsulinemia, ER stress, β-cell loss, misfolded insulin, and defects in the secretory pathway causing accumulation of proinsulin in the ER [16]. Wolcott-Rallison syndrome in humans [19] is characterized by infantile diabetes with defects in insulin secretion and folding [17, 32]. Mutations in the human PERK-encoding *Eif2ak3* gene were reported in two families with the syndrome [19].

In contrast, the role of IRE1α in β-cell and insulin biogenesis is less established. IRE1α is involved in β-cell failure in the Wolfram syndrome, also known as DIDMOAD (diabetes insipidus, diabetes mellitus, optic atrophy, and deafness) [33]. The Wolfram syndrome 1 gene (*Wfs1*) encodes an ER-resident protein associated with protein misfolding and β-cell failure. As XBP1s activates the *Wfs1* promoter, the IRE1α-XBP1s-WFS1 pathway represents a direct link between protein misfolding in the ER, the UPR, β-cell breakdown, and a diabetic patient cohort [34]. In transgenic mice, *Xbp1* deletion in β-cells impaired proinsulin processing, blunted glucose-stimulated insulin secretion, and caused IRE1α hyperactivation [35]. In turn, an overactive RIDD cleaved prohormone convertase mRNA [35], interfering in insulin maturation and secretion. Under hyperglycemic conditions, the RIDD can cleave insulin mRNA and reduce insulin production [36, 37]. Such signaling nodes may participate in our models.

At the ER membrane, IRE1α activity is tightly regulated by the UPRosome [38] involving UPR transducers, scaffold proteins, phosphatases, and ER-bound RNAs that physically bind to IRE1α in response to ER stress. IRE1α may cleave ER-bound RNAs, such BI-1 [39], strengthening the concept that BI-1 acts as a rheostat in diabetes. In addition, PDIA1, which supports proinsulin maturation [40], or changes in luminal acidification [41] may be specifically implicated, considering BI-1 activity can be regulated in acidification conditions [42]. Furthermore, the presence of rod-like granules [27, 28] in *BI-1-*deficient mice may be due to impaired calcium homeostasis causing insulin crystallization defects [28], since BI-1 has a significant role in maintaining Ca^2+^ homeostasis [43, 44]. Finally, increased insulin granule size may contribute to metabolic disorders by impairing their membrane trafficking at the trans-Golgi network (TGN) [45, 46], which could be involved in *BI-1*-deficient β-cells.

Our current data reveal that unresolved ER stress triggers a terminal UPR with sustained activity of IRE1α-XBP1-CHOP signaling that promotes programmed cell death, leading to pancreatic injury [47] and diabetes in *BI-1-*deficient mice. CHOP expression correlated with increased Puma and decreased BCL2 to promote apoptosis. This triggered mitochondrial dysfunction, shown by TEM analysis, as a downstream consequence of ER stress. The induction of JNKs, mediated by an overactive IRE1α [48], may be a contributor to β-cell death, alongside these observations. We also cannot exclude a compensatory activation of ATF6α or PERK, given the slight increase in ATF4.

Besides its cytoprotective role upon ER stress [15], our results suggest that BI-1 confers protection against IRE1α-induced caspase-1-dependent pyroptosis in β-cells. Further experiments in *BI-1*-deficient β-cells are needed to evaluate whether other genes are affected by the RNase activity of IRE1α, such as TXNIP, a contributor to prodiabetic pathways [49], which may amplify sterile inflammation and pyroptosis in our models. The IRE1α RNase inhibitor, STF-083010, improved pancreatic injury of the *BI-1*^–/–^ mice that recapitulated features of diabetes. The pancreatic inflammation observed in ND- and HFD-fed *BI-1*^–/–^ mice could be a consequence of extensive tissue injury, resulting in the release of pancreatic lipase, necrosis, and TUNEL-positive β-cells. Sustained NLRP3 inflammasome activation with infiltration of neutrophils was reported in diabetes [7, 49], in line with the observation that *BI-1*^–/–^ mice presented more MPO-positive cells in pancreatic sections that were corrected with IRE1α RNase inhibition. Since pharmacological targeting of the NLRP3 inflammasome alleviates pancreatic inflammation and β-cell death in preclinical models [50–53], small molecules targeting IRE1α RNase activity and/or the NLRP3 inflammasome could be attractive strategies in diabetes and chronic metabolic diseases. Our results strongly identify a novel protective role for BI-1 in pancreatic β-cell physiology as a stress integrator in β-cell function to modulate the UPR when faced with unfolded proinsulin accumulation, autophagy, and programmed cell death.

## Materials and methods

### Animal experimentation

*BI-1*^*+/+*^ (WT) and *BI-1*^*−/−*^ were obtained from Dr. John C. Reed (SBMRI, La Jolla, CA, USA) on a C57BL/6 background by disruption of the *bi-1* gene. Experiments were done on male mice at 8-week-old, 12-week-old, and 24-week-old mice. Mice were fed a Normal Diet (ND, A04–SafeDiet, Augy, France) or High-Fat Diet (HFD, 60% kJ fat, D12492 – sniff, Soest, Germany) and treated with intraperitoneal injections of STF-083010 (30 mg/kg) or vehicle (Kolliphor 16%). Mice were housed in a controlled environment with 12 h light/dark cycles and water available ad libitum.

### Ethics statement

Animal procedures were conducted in compliance with the French national (MESR, Ministère de l’Enseignement Supérieur, de la Recherche et de l’Innovation) guidelines for the use of experimental animals.

### Histological evaluation

Pancreas tissue was fixed in 10% buffered formalin, embedded in paraffin, sectioned (7 μm thick), and stained with H&E, Insulin, Glucagon, MPO, or TUNEL (Roche Molecular Biochemicals, Meylan, France). Sections were evaluated with bright-field microscopy.

### Electron microscopy

Pancreas was dissected, immersed in fixative 2.5% glutaraldehyde in 0.1 M cacodylate buffer, and stored overnight at 4 °C. Samples were rinsed in the same buffer, post-fixed for 1 h in 1% osmium tetroxide and 1% potassium ferrocyanide on 0.1 M cacodylate buffer to enhance the staining of membranes. Cells were then rinsed in distilled water, dehydrated in acetone at low temperature to preserve lipids and lastly embedded in epoxy resin. Contrasted ultrathin sections (70 nm) were analysed under a JEOL 1400 transmission electron microscope equipped with a Morada Olympus CCD camera. IMOD software was used to analyse images and delineate major cellular structures.

Immunogold labeling was done on epoxy sections after treatment with Sodium metaperiodate 5% in distilled water for 10 min., washes in H_2_O, followed by incubation in NH_4_Cl 50 mM in PBS, for 10 min., BSA1% in PBS for 15 min., and an overnight incubation with the primary antibody against insulin (Euromedex ref 158-48-1). After washes in PBS-BSA 0,1%, the PAG conjugated with 15 nm gold particle was added for 1 h, rinsed, briefly fixed 5 minutes with glutaraldehyde 1% in phosphate buffer, and finally sections were briefly contrasted with uranyl acetate.

### Cellular models

#### Islet isolation, glucose-stimulated insulin secretion, and islet viability

For islet isolation, mouse pancreases were digested by collagenase and incubated in a water bath at 37 °C. The islets were handpicked under a stereomicroscope. The isolated islets were cultured and treated as described. Glucose-stimulated insulin secretion (GSIS) was performed in freshly isolated islets Insulin was quantified using the Ultra-Sensitive Mouse Insulin ELISA Kit (Crystal Chem, Downers Grove, USA). The GSIS experiments were performed and measured in triplicates.

The percentages of viable cells were determined using the DNA-binding dyes Propidium Iodide (PI, 5 µg/mL, Sigma-Aldrich) and Hoechst 33342 (HO, 5 µg/mL, Sigma-Aldrich), as described [54]. The percentages of dead islets β-cells were evaluated in a minimum of 10 islets per condition. All assessments were performed by two independent researchers, including one in a blind manner.

#### Culture and transfection of EndoC-βH1 and cell viability

EndoC-βH1 cells were purchased from UNIVERCELL-BIOSOLUTIONS (MTA BH1-201601171) and cultured in low-glucose DMEM supplemented with 2% BSA fraction V, β-mercaptoethanol 50 µM, L-glutamine 1%, penicillin/streptomycin 2%, nicotinamide 10 mM, human transferrin 5.5 µg/ml and sodium selenite 6.7 ng/ml (all from Sigma-Aldrich, Diegem, Belgium) [54, 55]. Transfections of small interfering (si)RNAs (30 nmol/L, siBI-1 : HSS110620 *Invitrogen*) were performed using lipofectamine RNAimax (Fisher Scientific, Aalst, Belgium).

After transfection, the cells were then treated with the indicated concentrations of z-VAD-FMK (50 μmol/L, 30 min pre-incubation, Selleck, Munich, Germany), Nec-1 (10 μmol/L, MedChemExpress, Sollentuna, Sweden), Thapsigargin (1 μM, Sigma-Aldrich), or an equal volume of DMSO (Sigma-Aldrich), respectively, as specified. For viability analysis, EndoC-βH1were stimulated for 24-48 h.

EndoC-βH1 cell viability was measured by integration of viability dye (SYTOX™ Green, Invitrogen) on a Perkin Elmer VICTOR X3. The cells were treated as described. 5 mM SYTOX Green was added to cells, and the induced intensity was measured. The maximal fluorescence is obtained by full permeabilization of the cells by using Triton X-100 at a final concentration of 0.1%. This reported cell death as a percent of maximal SYTOX Green fluorescence (excitation 485 nm, emission 520 nm) from control conditions. The percentage of cell death was calculated as (induced fluorescence–background fluorescence)/(maximal fluorescence–background fluorescence) × 100. In some cases, percentage of cell death was normalized by the control condition siCtrl treated with DMSO.

#### Culture and transfection of MIN6 and cell death

MIN6 were obtained from the A.T.C.C. MIN6 were cultured in DMEM-High Glucose medium, 15% FBS, 1% P/S, 1% Glutamine (2 mM), and 50–55 μM beta-mercaptoethanol (1.75–2.0 μl/500 ml of medium). The cells were transfected with BI-1 siRNA (MSS272838) or control siRNA (Invitrogen, Medium) at 60 nM using Lipofectamine RNAiMAX (Invitrogen) in OptiMEM (Invitrogen) according to the manufacturer’s instructions. After 48 h of transfection, the cells were then treated with the indicated concentrations of Thapsigargin (5 μM, Sigma-Aldrich), Tunicamycin (5ug/ml), QVD-fmk (20 μM) YVAD-fmk (20 μM) (Peptide Institute, Osaka, Japan), or an equal volume of DMSO (Sigma-Aldrich), respectively, as specified.

Cell death was analyzed using a double fluorescent staining annexin-V-PE and 7-AAD according to the manufacturer’s instructions (Annexin-V-PE apoptosis detection kit I, BD Biosciences, Pont de claix, France). Single cell fluorescence was analyzed by flow cytometry (BD Canto II) and FlowJo software® (BD). The percentage of cell death (early and late apoptosis/death) was assessed (Annexin-V^+^/7-ADD^-^ and Annexin-V^+^/7-ADD^+^).

### Real-time qPCR

Total RNA was extracted from isolated pancreatic islets or cell lines (Endo-βH1 and MIN6), then reverse-transcribed for real-time quantitative PCR (RT-qPCR). RT-qPCR was performed using the ABI PRISM 7500/Step-One Fast Real-Time PCR System following the manufacturer’s protocols. Taqman gene expression assays purchased from Applied Biosystems (Courtaboeuf, France). *Mouse :* RPLP0 Mm99999223_gH ; BI-1 Mm00509863_m1 ; BIP Mm00517691_m1 ; CHOP Mm00492097_m1 ; XBP1 Mm00457359_m1. *Human :* RPLP0 Hs99999902_m1 ; BI-1 Hs00162661_m1 ; BIP Hs99999174_m1 ; CHOP Hs01090850_m1 ; XBP1 Hs00231936_m1. Gene expression values were normalized to the value of the housekeeping genes RPLP0 (Mm99999223_gH; human: Hs99999902_m1) or β2- microglobulin (Mm00437762_m1) and calculated based on the comparative cycle threshold Ct method (ΔΔCt).

### Immunoblot analysis

Total protein was isolated from snap-frozen pancreas, isolated pancreatic islets or cell lines homogenized in detergent-containing buffer, normalized for protein content (30 µg/tissue and 20 µg/cell sample), and analysed by SDS-PAGE (8–15% gels).

Antibodies references: p-IRE1 : NB100–2323 ; sXBP1 (mouse) : 7160 s ; sXBP1 (human) : CS127825 ; ATF4 : sc200 ; HSP90 : 4877 S ; NLRP3 : AG-20B-0014 ; IL-1b : 2021 S ; Caspase-1 : sc-514 ; tIRE1 CS-23945 ; p-PERK : cs-3179 ; Caspase-3 : cs96625 ; Bcl2 : cs2870 ; PUMA : ab-54288 ; LC3 : NB100-220 ; p62 : 5114 S ; ATG5/ATG12 complex : 26305 ; Ub K63 : ab179434 ; Parkin : CS-2132 ; COX4 : CS-4844.

Antibody detection was accomplished using horseradish peroxidase-conjugated secondary antibodies: Anti-Rabbit: 711-035-152 (Jackson) ; Anti-Mouse: 715-035-150 (Jackson).

The Image J software was used to measure band intensities from the immunoblots. To appreciate protein expression in pancreases, quantification was performed from several individuals for each genotype, as mentioned in the figure legend. The quantification analysis was performed from band intensities and expressed as fold change. The corresponding average quantification of the protein target compared with HSP90 levels or red-ponceau (as a loading control) was performed.

### Biochemical analysis and cytokine measurement

The BD Cytometric Bead Array Mouse Inflammation Kit was used to quantitatively measure cytokines by flow cytometry as described previously [10]. Serum triglyceride and cholesterol levels were determined by enzymatic colorimetric assay (Roche – Hitachi analyser Cobas 8000, Meylan, France). NEFA were measured using the Kit NEFA-HR (2) R1 Set (434-91795 from Fujifilm).

### Statistical analysis

Data are expressed as means ± SEM and were analyzed using GraphPad Prism 9 software.

Shapiro–Wilk normality test was performed to confirm the normal distribution of the data. Statistical significance of differential gene expression between the two study groups was determined using the non-parametric Mann–Whitney test, with the ΔCt of each group. Other data from mice and cells were statistically analysed by Student’s *t* test, Mann–Whitney or one-way ANOVA and *post hoc* analysis for multiple group comparison. Statistical significance from control is denoted by *P ≤ 0.05; **P ≤ 0.01; ***P ≤ 0.001. ****P ≤ 0.0001. Following the same pattern, $ or # or £ denotes statistical significance between specified groups.

### Supplementary information

Supplemental figures and legends

Supp Original data file

## Data Availability

All data needed to evaluate the conclusions in the paper are present in the paper. Additional data related to this paper may be requested from the corresponding author.
