# Supplementary material for: Bax Inhibitor-1 preserves pancreatic β-cell proteostasis by limiting proinsulin misfolding and programmed cell death
Source: Cell Death Dis. 2024 May 14;15(5):334. doi: 10.1038/s41419-024-06701-x (PMC11094198; doi:10.1038/s41419-024-06701-x)
Supplement: Supplementary file 1 — Supplemental figures and legends [file 41419_2024_6701_MOESM1_ESM.pdf]

Figure S1

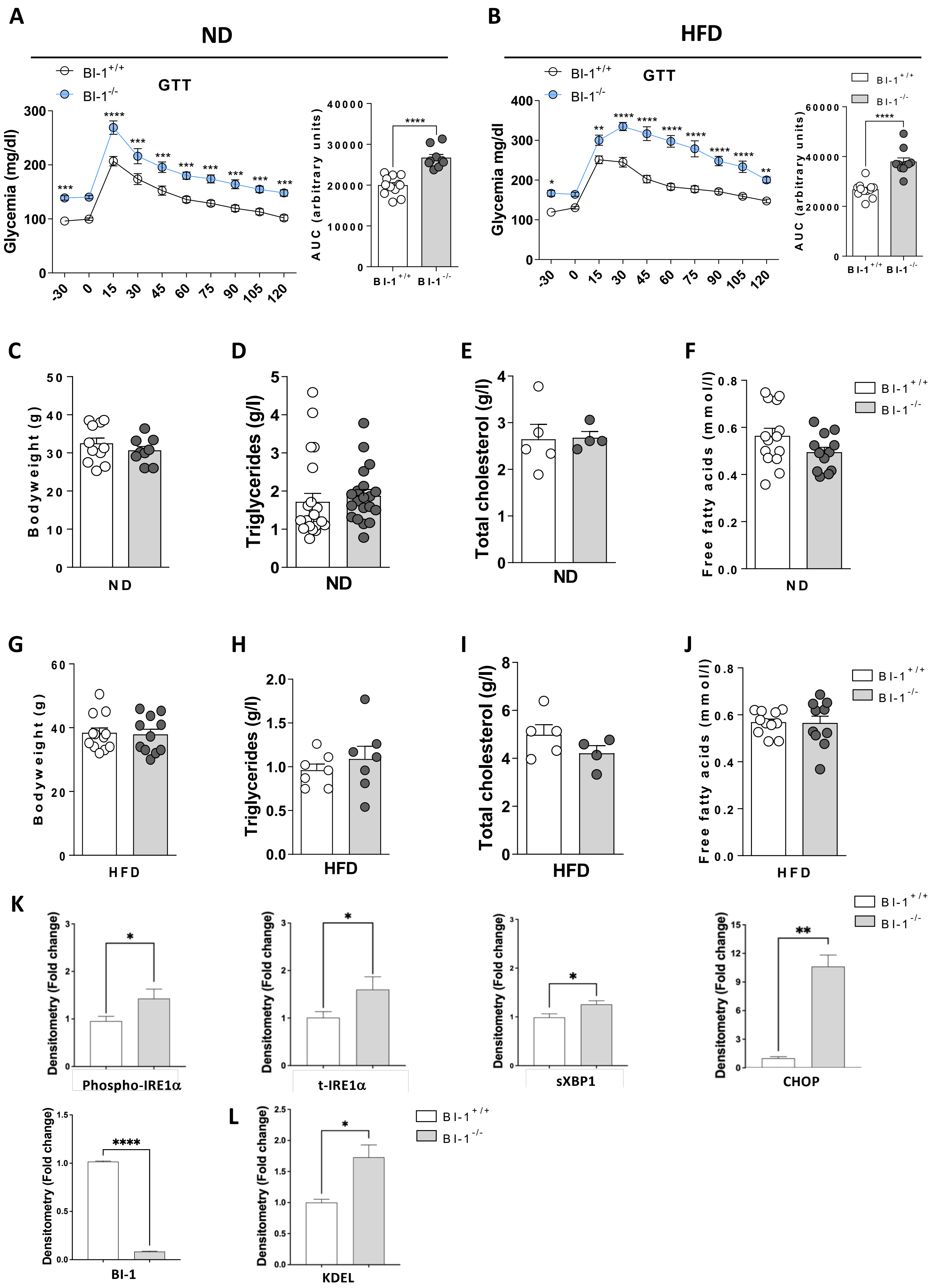

A
ND
B
HFD
GTT
BI-1+/+
BI-1-/-
Glycemia (mg/dl)
Glycemia mg/dl
AUC (arbitrary units)
C
D
E
F
Bodyweight (g)
Triglycerides (g/l)
Total cholesterol (g/l)
Free fatty acids (mmol/l)
ND
G
H
I
J
HFD
K
Densitometry (Fold change)
Phospho-IRE1α
t-IRE1α
sXBP1
CHOP
BI-1
L
KDEL

Figure S2

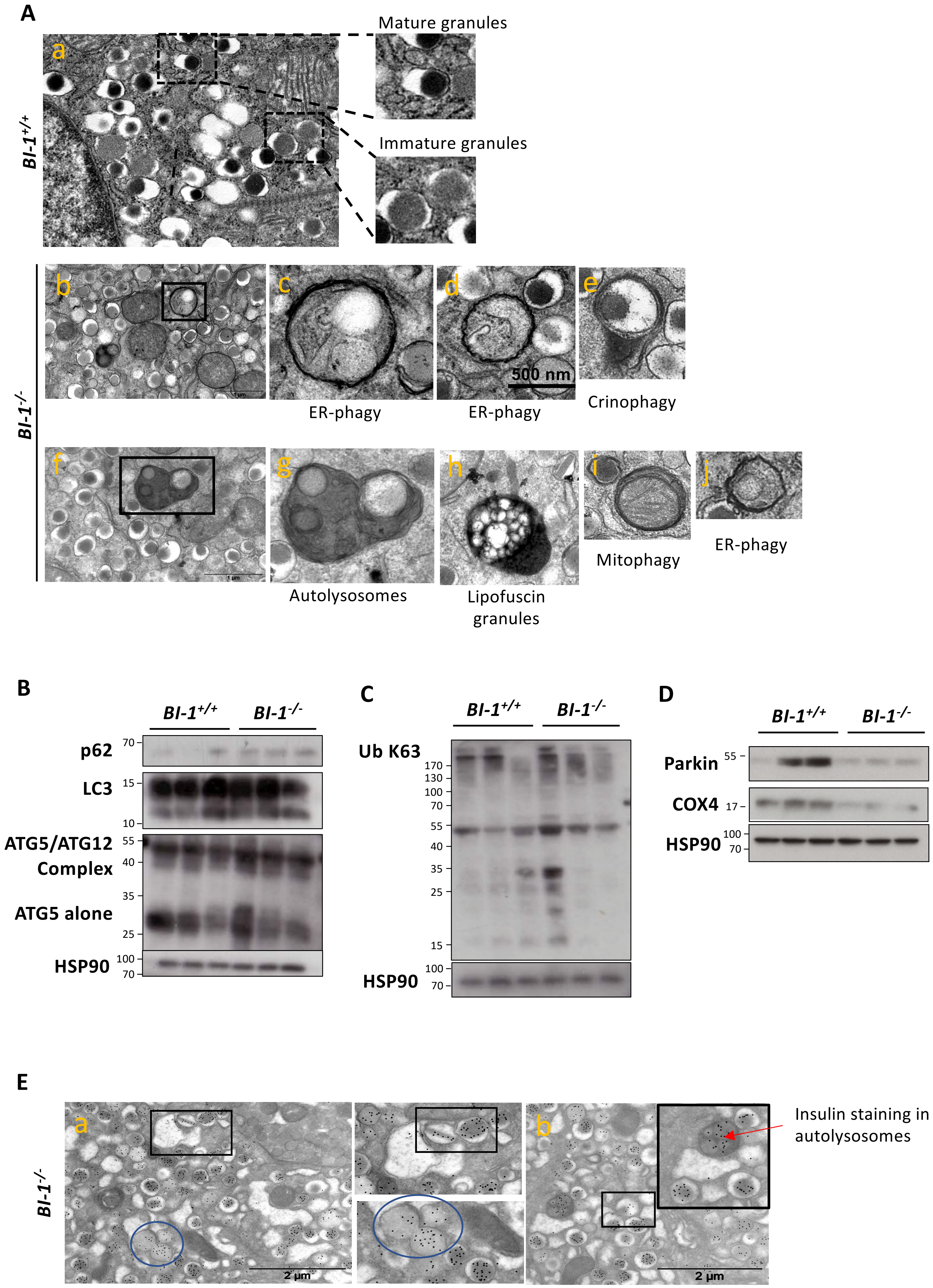
A
a
Mature granules
Immature granules
BI-1+/+
BI-1-/-
b
c
d
e
500 nm
ER-phagy
ER-phagy
Crinophagy
f
g
h
i
j
Autolysosomes
Lipofuscin granules
Mitophagy
ER-phagy
B
BI-1+/+
BI-1-/-
p62
LC3
ATG5/ATG12 Complex
ATG5 alone
HSP90
70
15
10
55
40
35
25
100
70
C
BI-1+/+
BI-1-/-
Ub K63
170
130
100
70
55
40
35
25
15
HSP90
100
70
D
BI-1+/+
BI-1-/-
Parkin
COX4
HSP90
55
17
100
70
E
BI-1-/-
a
b
2 µm
2 µm
Insulin staining in autolysosomes

Figure S3

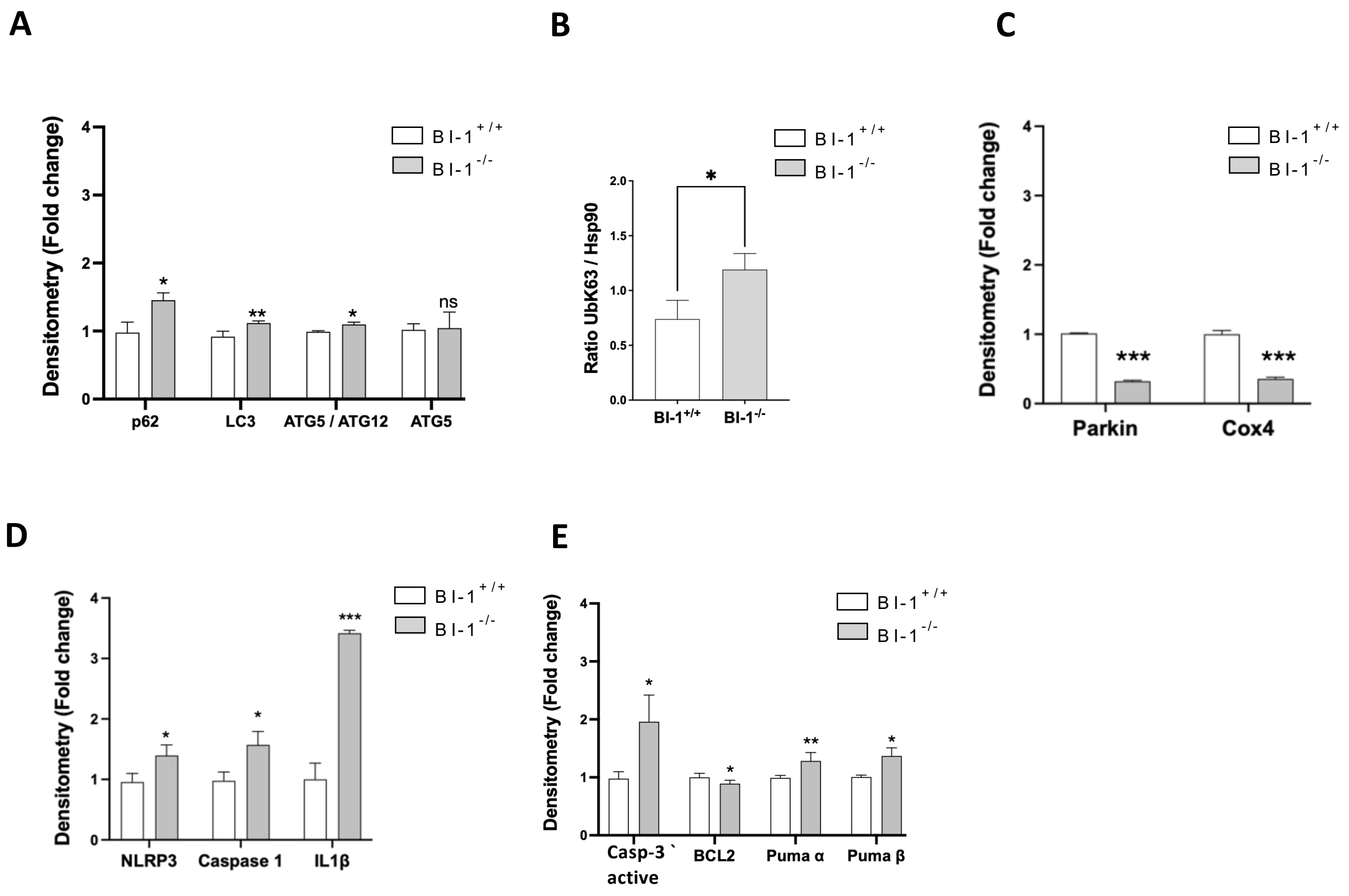
A
B
C
D
E
Densitometry (Fold change)
B I-1 +/+
B I-1 -/-
p62
LC3
ATG5 / ATG12
ATG5
ns
Ratio UbK63 / Hsp90
BI-1+/+
BI-1-/-
Parkin
Cox4
NLRP3
Caspase 1
IL1β
Casp-3 ` active
BCL2
Puma α
Puma β

Figure S4

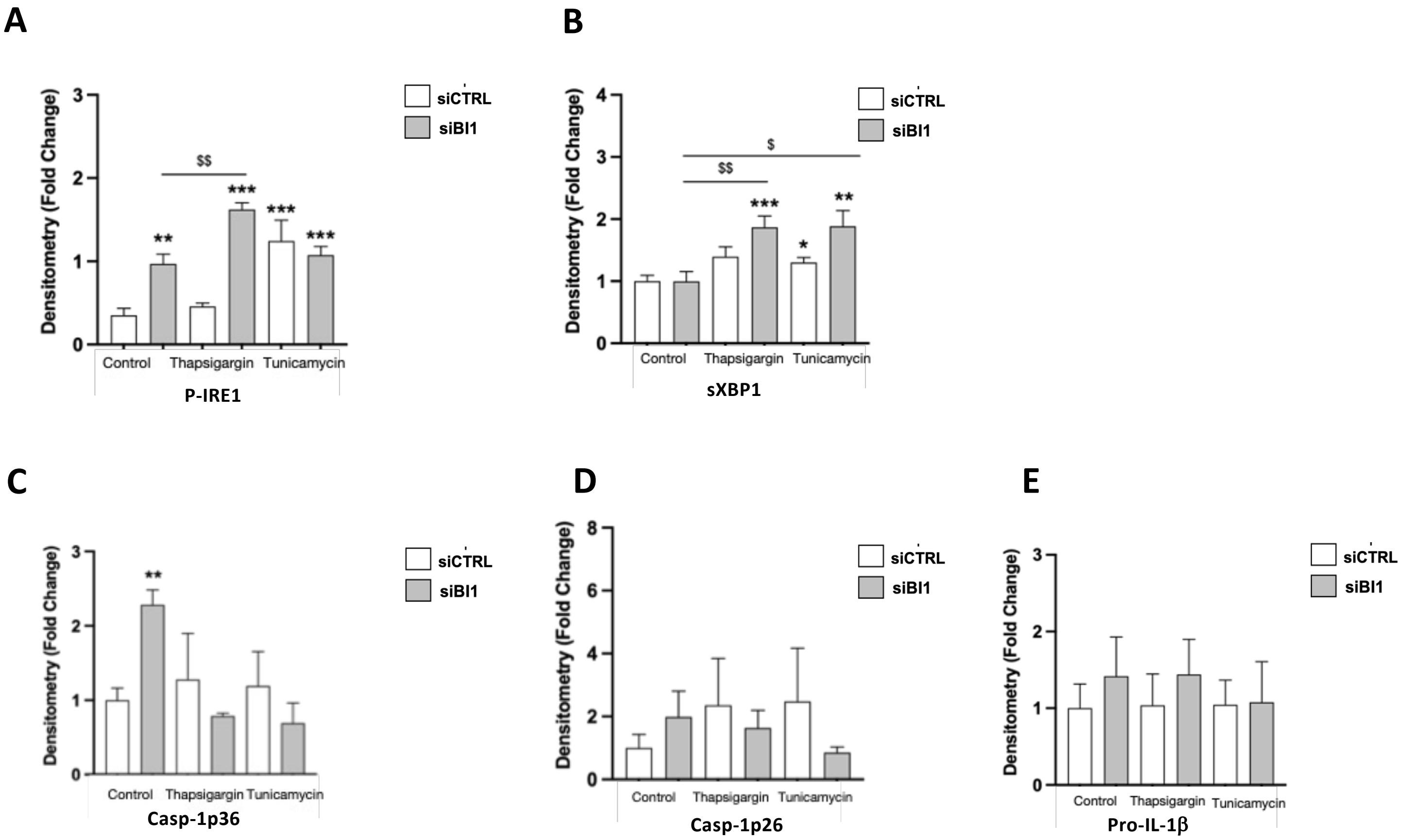
A
B
C
D
E
Densitometry (Fold Change)
siCTRL
siBI1
Control
Thapsigargin
Tunicamycin
P-IRE1
sXBP1
Casp-1p36
Casp-1p26
Pro-IL-1β
$$
$
***
**
*

Figure S5

A

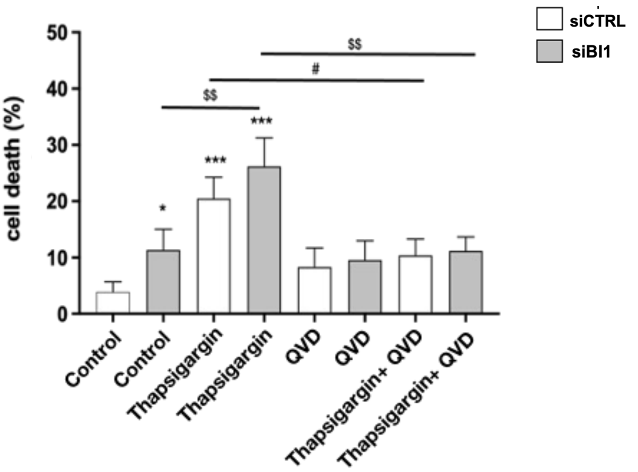

B

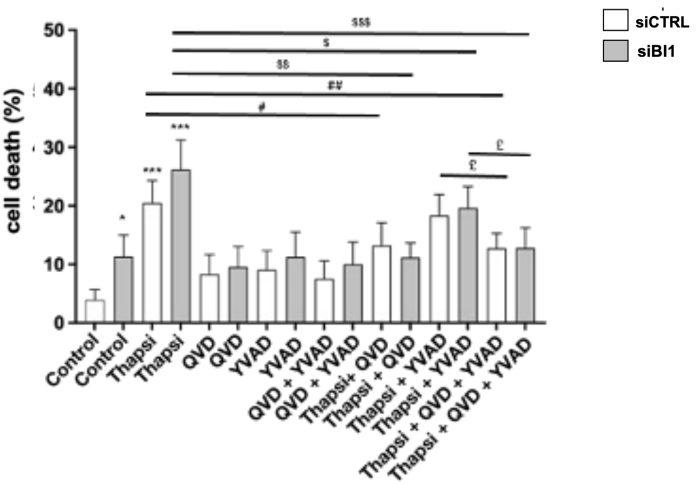

C

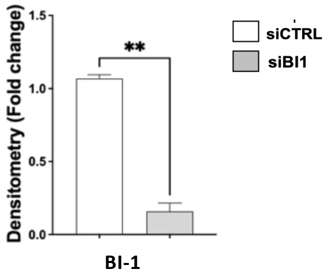

D

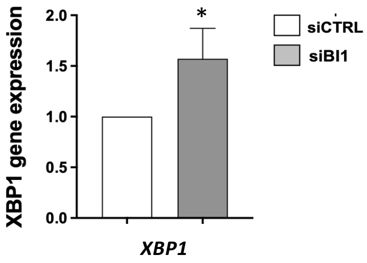

Figure S6

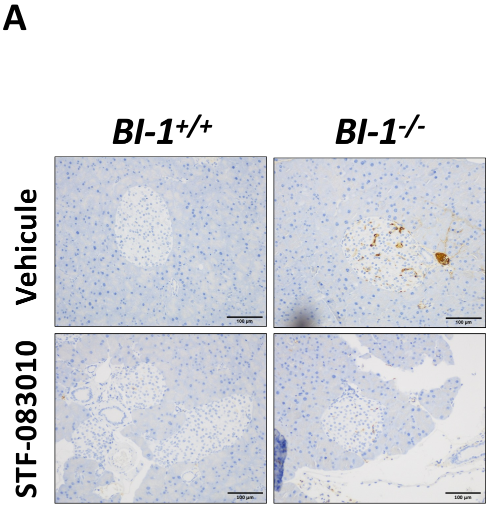
A
BI-1+/+
BI-1-/-
Vehicule
STF-083010

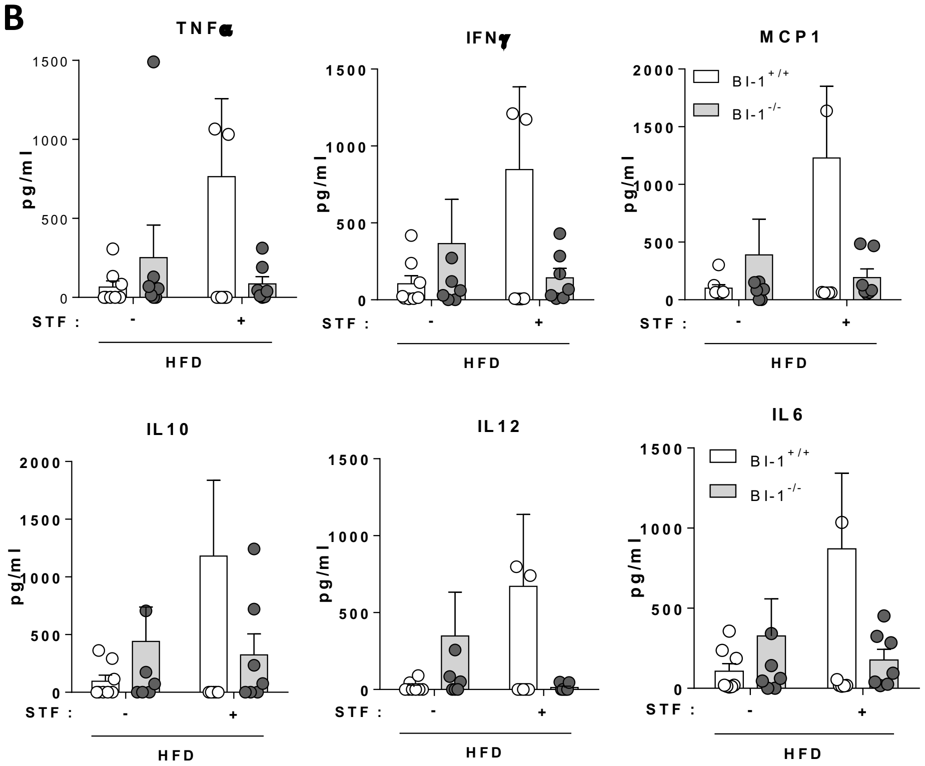
B
TNFα
IFNγ
MCP1
IL10
IL12
IL6
pg/ml
STF :
-
+
HFD
BI-1+/+
BI-1-/-

Supplementary Figures

Figure S1. Related to Figure 1. ***BI-1 deletion leads to glucose intolerance but does not affect circulating lipid levels and energy expenditure.*** *BI-1*$^{+/+}$ and *BI-1*$^{-/-}$ mice were fed a normal diet (ND) for 6 months or a 3-month HFD starting at 3 months old. **(A)** Glucose Tolerance Test (GTT) of fasted *BI-1*$^{+/+}$ and *BI-1*$^{-/-}$ mice under ND were performed, Area Under the Curve (AUC) is presented. *n* = 9 – 12 mice per group. **(B)** Glucose Tolerance Test (GTT) of fasted *BI-1*$^{+/+}$ and *BI-1*$^{-/-}$ mice under a 3-month HFD were performed, Area Under the Curve (AUC) is presented. *n* = 9 – 12 mice per group. On ND fed mice, **(C)** bodyweights were measured. *n* = 9 - 11 mice. **(D)** Serum triglycerides levels were measured. *n* = 20 mice per group. **(E)** Serum cholesterol levels are shown. *n* = 4 – 5 mice per group. **(F)** Serum free fatty acids levels were analyzed. *n* = 12 – 14 mice per group. On 3-month HFD fed mice, **(G)** bodyweights were measured. *n* = 9 - 11 mice. **(H)** Serum triglycerides levels were measured. *n* = 7 mice per group**.** **(I)** Serum cholesterol levels are shown. *n* = 4 – 5 mice per group**.** **(J)** Serum free fatty acids levels were analyzed. *n* = 10 – 12 mice per group**.** **(K)** Average quantification of phospho-IRE1α, tIRE1α, sXBP1, CHOP and BI-1 compared with B-actin levels (used as a loading control) **(L)** KDEL (PDI) is shown compared with loading control level (red ponceau) n = 6 per genotype**.** *P ≤ 0,05; **P ≤ 0,01; ***P ≤ 0,001. **** P ≤ 0,0001

Figure S2. Related to Figure 2. ***Lack of BI-1 favours autophagy, inflammation, and pancreatic injury.***

BI-1$^{+/+}$ and *BI-1*$^{-/-}$ mice were fed a normal diet (ND) for 6 months. **(A)** Analysis of insulin granules and vesicular structures by TEM with morphologies resembling autophagic vesicles [Scale bar, 1μm]. Pictures (a) mature and immature insulin granules, (b, c, d, f, g) *BI-1*$^{-/-}$ pancreatic sections showing autophagic vesicles containing ER (b, c, d, j), insulin granules (f, g), mitochondria (i), (h) RB-L residual body or Lipofuscin lysosomal granule. **(B)**

Immunoblotting analysis of pancreatic autophagy protein readouts from *BI-1*$^{+/+}$ and *BI-1*$^{-/-}$. *n* = 3 mice per group. **(C)** Immunoblotting analysis of K63 ubiquitination in total pancreas. *n* = 3 mice per group. **(D)** Immunoblotting analysis of Parkin and COX4 in total pancreas. *n* = 3 mice per genotype. **(E)** On insulin immunogold staining, we observed stained granules in autolysosomes (in b). ER shows limited gold particles even if the antibody stains immature insulin.

Figure S3. Western-blotting quantifications Related to Figure S2. (A) Average quantification of p62, LC3, ATG5/ATG12, ATG5 **(B)** Ub K63 **(C)** Parkin, COX4. **Western-blotting quantifications Related to Figure 3A and Figure 3E. (D)** NLRP3, Capase-1, IL-1β, CHOP and **(E)** Active Caspase-3, BCL2, Puma α, Puma β compared with HSP90 levels (used as a loading control). n = 6 per genotype. *P ≤ 0,05; **P ≤ 0,01.

Figure S4. Western-blotting quantifications Related to Figure 5C. (A-E) Phospho-IRE1α, XBP1s, Casp-1p36, Casp-1p26 and pro-IL-1β. (n = 4-7 independent experiments.) *P ≤ 0,05; **P ≤ 0,01.

Figure S5. ***BI-1 deletion makes more vulnerable murine β-cell to ER stress induced apoptosis and inflammasome activation.*** **(A)** Cell death was quantified in the murine β−cell line MIN6. Cells were pretreated with q-VD-fmk (QVD, 20 μM) for 1 h prior to being treated with chemical ER stress thapsigragin (5 μM) for 24 h compared to normal media. The percentage of cell death was quantified by flow cytometry. *n* = 10-12 per conditions. *P ≤ 0,05; **P ≤ 0,01; ***P ≤ 0,001. * Represents differences with control**.** $ and # represents differences with indicated treated conditions**.** $$ P ≤ 0,01. # P ≤ 0,05. **(B)** Cell death was quantified in MIN6. Cells were pretreated with YVAD-fmk (YVAD, 20 μM) for 1 h prior to being treated with chemical ER

stress thapsigragin (5 μM) for 24 h compared to normal media. **(C)** Average quantification of BI-1 protein compared with HSP90 levels (used as a loading control) (n = 3 independent experiments). **(D)** qPCR analysis of *xbp1* gene (n = 3 independent experiments). *P ≤ 0,05; **P ≤ 0,01.

Figure S6. ***STF-083010 treatments correct inflammation in BI-1 deficient mice.***

(A) Representative images of myeloperoxidase (MPO) staining from *BI-1*$^{+/+}$ and *BI-1*$^{-/-}$ pancreatic sections injected with STF-083010 or vehicle. *n* = 3 mice per group. [Scale bar, 100 μm**]** **(B)** Relative serum cytokines levels evaluated by flow cytometry analysis. *n* = 8 mice per Group.
