# Supplementary material for: Bax Inhibitor-1 preserves pancreatic β-cell proteostasis by limiting proinsulin misfolding and programmed cell death
Source: Cell Death Dis. 2024 May 14;15(5):334. doi: 10.1038/s41419-024-06701-x (PMC11094198; doi:10.1038/s41419-024-06701-x)

FIGURE 1E

Phospho-IRE1

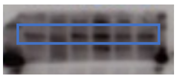

t-IRE1

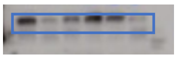

Xbp1

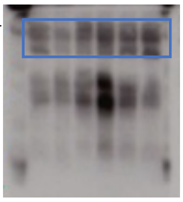

CHOP

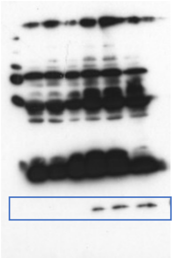

BI-1

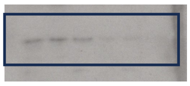

β-Actin

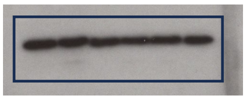

FIGURE 1G

KDEL

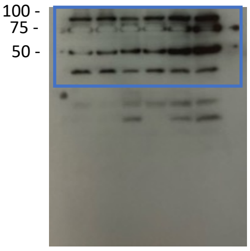

Loading control red ponceau

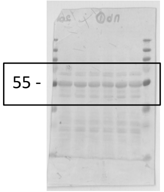

Figure 3 A

NLRP3

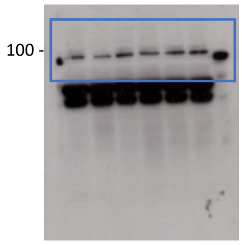

Caspase 1 p26

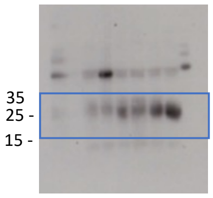

IL-1bp17

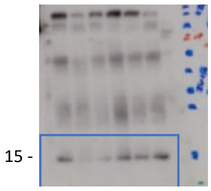

HSP90

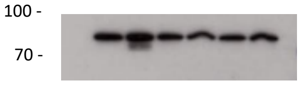

Figure 3E

Caspase-3

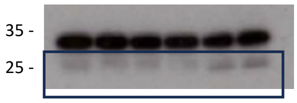

Bcl2

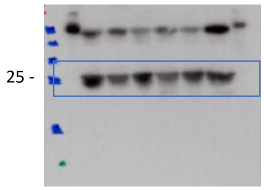

Puma α et β

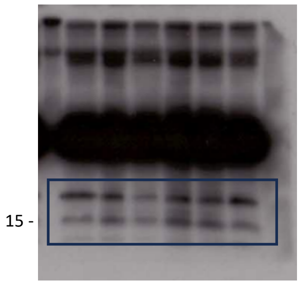

HSP90

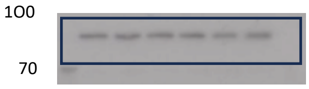

Figure 4 F

Proinsuline

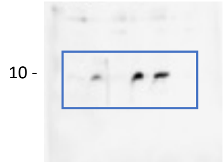

Loading control (ponceau red)

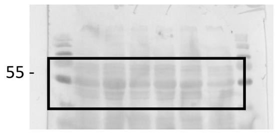

Figure 5 C

p-IRE1

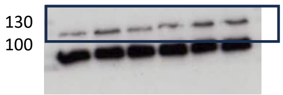

total IRE1

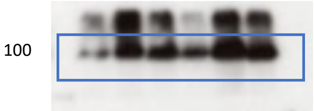

sXBP1

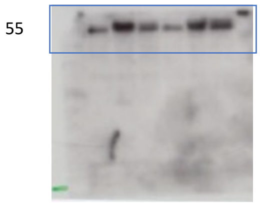

Caspase 1

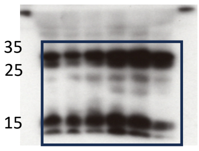

Pro-IL-1β

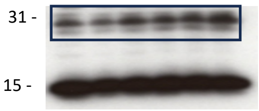

HSP90

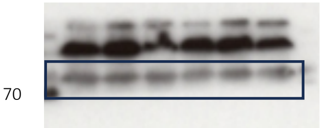

Figure S2B

P62

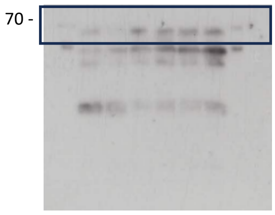

LC3

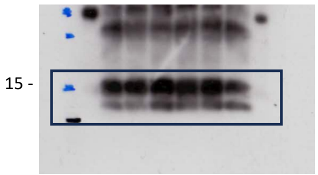

ATG5/ATG12 complexe et ATG5 alone

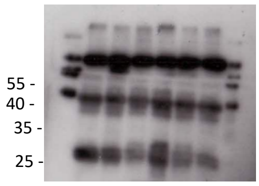

HSP90

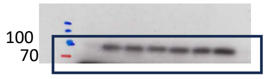

Figure S2C

UbK63

170

55

15

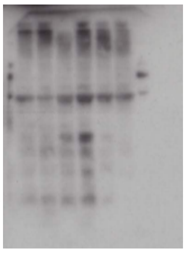

Hsp90

100

70 -

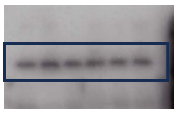

Figure S2D

Parkin

55 -

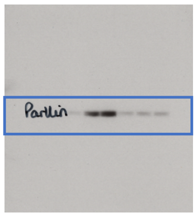

COX4

17 -

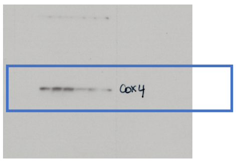

Hsp90

100

70 -

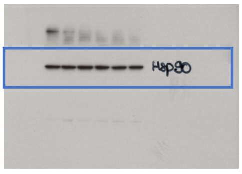

Supplement: Supplementary file 2 — Supp Original data file [file 41419_2024_6701_MOESM2_ESM.pdf]
